# Predicting admission for fall‐related injuries in older adults using artificial intelligence: A proof‐of‐concept study

**DOI:** 10.1111/ggi.15066

**Published:** 2025-01-12

**Authors:** Nam Le, Milan Sonka, Dionne A Skeete, Kathleen S Romanowski, Colette Galet

**Affiliations:** ^1^ Iowa Initiative for Artificial Intelligence University of Iowa Iowa City Iowa USA; ^2^ Department of Electrical and Computer Engineering University of Iowa Iowa City Iowa USA; ^3^ Division of Acute Care Surgery, Department of Surgery University of Iowa Roy J. and Lucille A. Carver College of Medicine Iowa City Iowa USA; ^4^ Division of Burn Surgery University of California, Davis Medical Center and Shriners Children's Northern California Sacramento California USA

**Keywords:** admission predictors, fall injury, machine learning, National Readmission Database, older adults

## Abstract

**Aim:**

Pre‐injury frailty has been investigated as a tool to predict outcomes of older trauma patients. Using artificial intelligence principles of machine learning, we aimed to identify a “signature” (combination of clinical variables) that could predict which older adults are at risk of fall‐related hospital admission. We hypothesized that frailty, measured using the 5‐item modified Frailty Index, could be utilized in combination with other factors as a predictor of admission for fall‐related injuries.

**Methods:**

The National Readmission Database was mined to identify factors associated with admission of older adults for fall‐related injuries. Older adults admitted for trauma‐related injuries from 2010 to 2014 were included. Age, sex, number of chronic conditions and past fall‐related admission, comorbidities, 5‐item modified Frailty Index, and medical insurance status were included in the analysis. Two machine learning models were selected among six tested models (logistic regression and random forest). Using a decision tree as a surrogate model for random forest, we extracted high‐risk combinations of factors associated with admission for fall‐related injury.

**Results:**

Our approach yielded 18 models. Being a woman was one of the factors most often associated with admission for fall‐related injuries. Frailty appeared in four of the 18 combinations. Being a woman, aged 65–74 years and presenting a 5‐item modified Frailty Index score >3 predicted admission for fall‐related injuries in 80.3% of this population.

**Conclusion:**

Using artificial intelligence principles of machine learning, we were able to develop 18 signatures allowing us to identify older adults at risk of admission for fall‐related injuries. Future studies using other databases, such as TQIP, are warranted to validate our high‐risk combination models. **Geriatr Gerontol Int 2025; 25: 232–242**.

## Graphical abstract

Using artificial intelligence principles of machine learning, the national readmission database was mined to identify factors, including frailty, associated with admission of older adults for fall‐related injuries. Our approach yielded 18 models. Being a woman, aged 65–74 years and frail predicted admission for fall‐related injuries in 80.3% of this population.

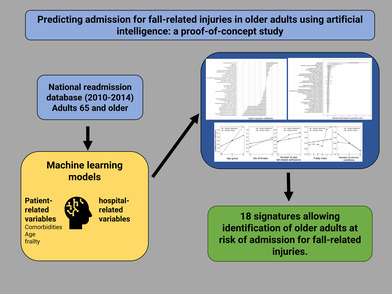

## Introduction

In addition to mortality and readmission risk, falls among older adults represent an economic burden. Falls result in approximately $50 billion in medical costs for non‐fatal fall injuries, and $754 million in costs related to fatal falls.^1^, ^2^ These data highlight the need for the development of fall risk screening tools to identify those older adults at risk for fall. Primary care providers can help mitigate fall risk by review and adjustment of high risk medications, and/or referring them to evidence‐based fall prevention programs or physical therapy.

Predicting medical outcomes using artificial intelligence principles of machine learning (AI/ML) to develop preventative and/or curative protocols has been achieved in many fields of medicine.^3^, ^4^, ^5^, ^6^ However, despite the fact that 2.8 million older adults (aged >65 years) are treated in emergency departments for fall injuries, representing 10–15% of all emergency department visits, fall‐related injuries lead to 800 000 hospitalizations a year.^7^, ^8^ Such approaches have not been frequently used when it comes to identifying factors associated with fall risks and hospital admission for fall‐related injuries in older adults. Only a few studies have looked at falls using AI/ML. Razmara *et al*. utilized artificial neural networks to predict fall risk in older adults.^9^ Lathouwers *et al*. used a ML approach to develop a fall‐risk classification algorithm identifying community‐dwelling older adults at higher risk of falling based on surveys including biological, behavioral, environmental and socio‐economic risk factors. They showed that maintaining a mental, physical and socially active lifestyle was associated with a reduced risk of falling.^10^ Despite these initial forays into using these AI/ML techniques to predict patients who will sustain falls, more work in this area is needed.

Several fall screening tools have been developed, some of which include frailty as a predictor.^11^ Frailty is defined as a multidimensional state of loss of physiological reserves, including energy, physical ability, cognition and health.^11^, ^12^, ^13^, ^14^, ^15^, ^16^ In fact, pre‐injury frailty has been investigated as a tool to predict outcomes of older surgical, trauma and burn patients.^17^, ^18^, ^19^, ^20^, ^21^ Multiple frailty assessments have been developed to try to determine a patient's functional status.^16^, ^22^, ^23^, ^24^ The 5‐item modified Frailty Index (mFI‐5) uses preoperative variables identified from the National Surgical Quality Improvement Project that were mapped from items of the Canadian Study of Health and Aging Frailty Index.^25^

In the present study, we used AI/ML to identify a “signature” (combination of clinical variables) that could predict which older adults are at risk for a fall and hospital admission for fall‐related injuries. To this end, the National Readmission Database (NRD) was mined to identify factors associated with admission of older adults for fall‐related injuries. We hypothesized that frailty, measured using the mFI‐5, could be utilized in combination with other factors as a predictor of admission for fall‐related injuries.

## Methods

### Ethics statement

This study was reviewed by our Institutional Review Board. It did not meet the regulatory definition of human subject research and did not require review, because the NRD only contains deidentified data (IRB# 201709803).

### Data source

The NRD is part of a family of databases and software tools developed for the Healthcare Cost and Utilization Project. The NRD are calendar‐year files based on discharge date for all data years, except 2015. The NRD contains clinical and nonclinical variables that support readmission analyses, with safeguards to protect the privacy of individual patients, physicians and hospitals. There is no data element identifying whether sequential inpatient stays are related or unrelated. The NRD is drawn from the Healthcare Cost and Utilization Project State Inpatient Databases (https://www.hcup-us.ahrq.gov/nrdoverview.jsp#about).

### Data description

The NRD data files from 2010 to 2014 were purchased from the Healthcare Cost and Utilization Project. The patient population was selected as previously described.^26^ Briefly, hospitalizations of older patients (aged ≥65 years) with a trauma principal diagnosis were our index events. Trauma patients were defined as those whose first five diagnosis codes (dx1–dx5) were International Classification of Diseases, Ninth Revision, Clinical Modification (ICD‐9) injury diagnoses 800–999 or any Ecode belonging to the following categories: E800–E849 or E880–E928, excluding patients with codes for superficial injury, foreign bodies or late effects of an injury. E‐code E‐885.0–E‐886.9 (same level falls), E‐880.0–E‐880.9 (fall from stairs or steps), E‐881.0–E‐884‐9 (fall from height) and E‐code 888.0–E‐888.9 (other or unspecified falls) were used to identify older trauma patients who suffered a fall. Overall, 4 939 303 were identified, 1 826 749 (37%) suffered a fall and 3 112 554 (63%) suffered non‐fall trauma. Age was categorized as 65–74, 75–84 and ≥85 years. The number of chronic conditions was grouped as follows: 1: 0–3 chronic conditions, 2: 4–11 and 3: ≥11 chronic conditions. The hospital length of stay was categorized as follows: 1: 1–5 days, 2: 6–10 days, 3: 3: 11–20 days, 4: 21–50 days, 5: 51–100 days and 6: ≥101 days. Because some patients experienced more than one hospital admission during the years 2010–2014, we summarized at the patient‐level the number of past hospital visits for traumatic injuries as follows: (i) zero past admissions, (ii) one past admission, and (iii) more than two past hospital admissions.

### Frailty scoring

Frailty scoring was carried out using the mFI‐5, as previously described.^27^, ^28^ Briefly, the mFI‐5 is based on the presence of each of the following comorbidities: hypertension requiring treatment (CM_HTN_C), diabetes mellitus (CM_DM, CM_DMCX), chronic obstructive pulmonary disease (CM_CHRNLUNG), congestive heart failure (CM_CH F) and impaired functional status. Impaired functional status was determined using the following ICD‐9 codes available in the NRD as surrogate measures of impaired functional status: V550‐V556; V559‐V562; V5489, V5849, V5632, V603, V4984, V4989, V6089, V604, V6089, V6149, V440‐V444, V4450‐V4452; V4459, V446, V449, V451, V461, V463 and V468. (Table S1).

**Figure 1:**
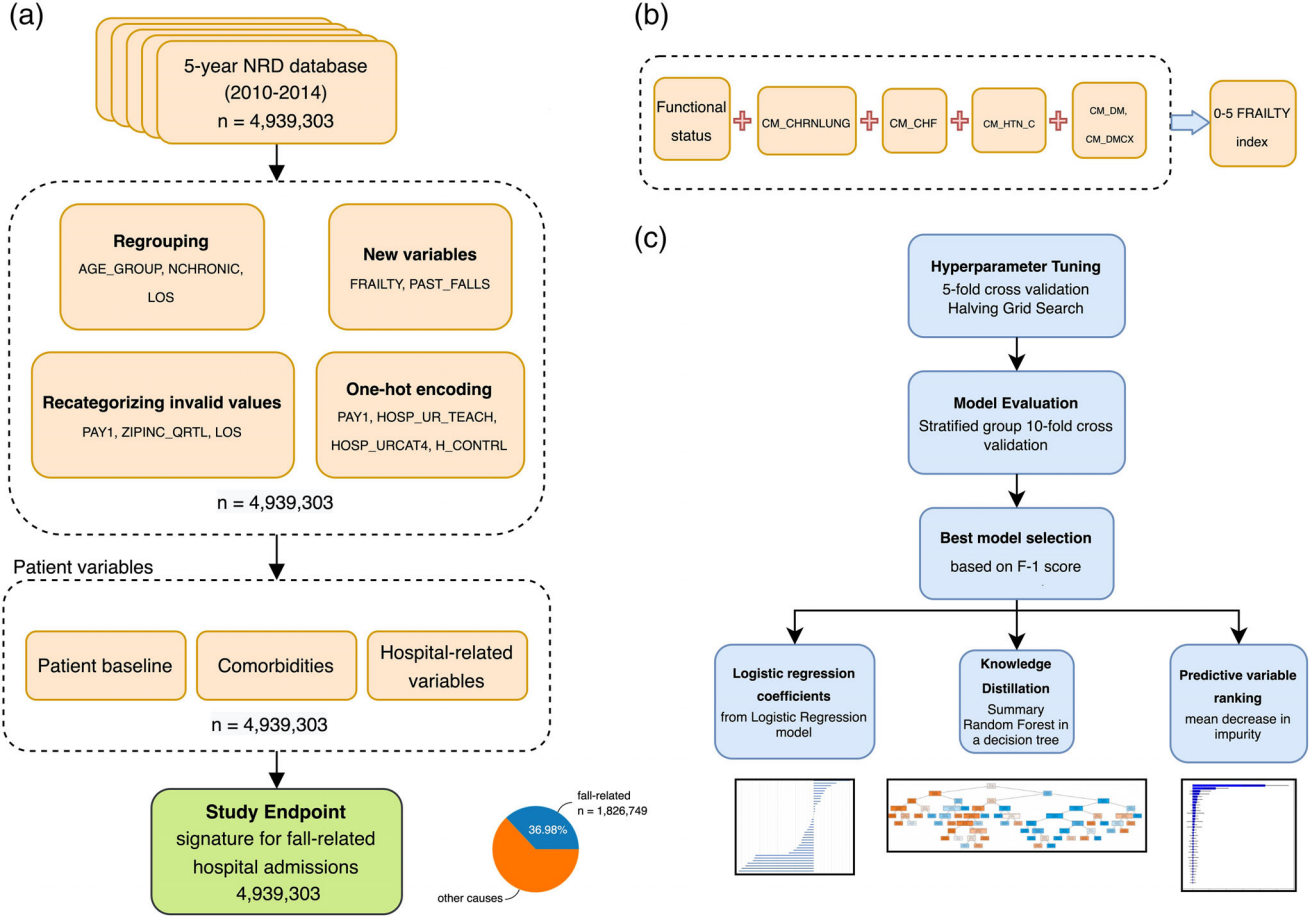
Schematic diagrams describing the process of data selection and machine learning model development. (a) Flow diagram showing steps in selecting data samples for training and testing, and preparing patient variables for study endpoints. (b) Construction of the frailty score. (c) The process of hyperparameter tuning and cross‐validation for machine learning model evaluation. NRD, National Readmission Database.

We scored patients based on the presence of each comorbidity (1 point) providing mFI‐5 scores ranking from 0 to 5. Based on the literature, patients presenting a score of ≥3 were considered severely frail (Fig. 1b).^27^, ^28^

### Primary and secondary endpoints

Our primary endpoint was to identify factors associated with admission of older adults for fall‐related injuries by mining the data elements available in the NRD. Our secondary endpoint was to determine whether frailty can be utilized in combination with other factors as a predictor of admission for fall‐related injuries.

### Data selection and machine learning model development

Data selection and machine learning model development are presented in Fig. 1. Problematic and missing NRD categories such as ‘A’, ‘C’, representing 0.102% of expected primary payer records were coded as numeric 0. Expected primary payors were one‐hot encoded and presented as seven single‐category variables describing types of medical‐bill payor. A total of 36 variables were studied for a population of 1 048 575 patients (Table S2). Class weighting was applied to address the class imbalance in the dataset. Collinearity between the number of chronic conditions, comorbid conditions and frailty was assessed. The variance inflation factors ranged from 1.00 to 1.69, showing no to extremely low collinearity.

### Machine learning model selection

As shown in Table S3, the prediction performance (F1 score) of six ML models – random forest classifier (RF), logistic regression classifier (LR), support vector machine, Multi‐layer perceptron classifier, decision tree and naïve Bayes classifier – was compared to select the two best‐performing ML models, which were used in the second stage to examine key predictors for patients admitted for a fall injury.^29^, ^30^, ^31^, ^32^, ^33^ To retain prediction interpretability, numerical variables, such as age group, number of chronic conditions, frailty index and number of past admissions, were not z‐normalized during the training and validation. The two best‐performing models based on F‐1 scores were RF and LR classifiers. Areas under the curve were similar for these two models, 0.693 and 0.688, respectively.

### Signature variable analysis

The most influential variables for the prediction of admission for fall‐related injury were evaluated for both the RF and LR models using 10% of the data. Partial dependence plot analysis showing the relationship of a variable and admission for fall‐related injury was carried out. Knowledge distillation was used to understand the complex decision‐making process of the RF model, yielding 18 high‐risk combinations of factors for fall‐related admissions.^34^

### Statistical analysis

The characteristics of patients admitted for a fall‐related injury (Fall group), as compared with those admitted for other trauma‐related injury (No fall group), are presented in Table 1. Model development was carried out in a Python 3.8.5 (Python Software Foundation, Beaverton, OR, USA) environment. Computing tools used included scikit‐learn 1.1.1 and pandas 1.4.2.^35^, ^36^ For the logistic regression model, coefficients, standard error and *P*‐values are provided.

**Table 1:** Characteristics of patients who were admitted to hospitals between 2010 and 2014 for a fall injury (fall group) versus for other causes (no fall group)

Variables/features	No fall group	Fall group	*P*‐value
	*n* = 667 726	*n* = 380 849	
Female, *n* (%)	351 080 (52.6)	258 619 (67.9)	<0.001
Age group 1 65–74 years, *n* (%)	283 901 (42.5)	85 161 (22.4)	<0.001
Age group 2 75–84 years, *n* (%)	249 841 (37.4)	249 841 (37.9)	
Age group 3 >85 years, *n* (%)	133 984 (20.1)	151 291 (39.7)	
Comorbidities			
AIDS, *n* (%)	225	76	<0.001
Alcohol abuse, *n* (%)	13 255 (2)	12 126 (3.2)	<0.001
Chronic blood loss anemia, *n* (%)	12 231 (1.8)	7057 (1.9)	0.437
Coagulopathy, *n* (%)	50 160 (7.5)	22 368 (5.9)	<0.001
Deficiency anemias, *n* (%)	170 427 (25.5)	97 847 (25.7)	0.058
Depression, *n* (%)	62 959 (9.4)	50 217 (13.2)	<0.001
Drug abuse, *n* (%)	5379 (0.8)	1868 (0.5)	<0.001
Fluid and electrolyte disorders, *n* (%)	226 559 (33.9)	115 875 (30.4)	<0.001
Hypothyroidism, *n* (%)	100 460 (15)	73 457 (19.3)	<0.001
Liver disease, *n* (%)	13 164 (2)	5382 (1.4)	<0.001
Lymphoma, *n* (%)	9841 (1.5)	3319 (0.9)	<0.001
Metastatic cancer, *n* (%)	24 673 (3.7)	5545 (1.5)	<0.001
Solid tumor without metastasis, *n* (%)	27 637 (4.1)	7830 (2.1)	<0.001
Obesity, *n* (%)	52 939 (7.9)	18 056 (4.7)	<0.001
Other neurological disorders, *n* (%)	74 690 (11.2)	57 394 (15.1)	<0.001
Paralysis, *n* (%)	25 368 (3.8)	12 230 (3.2)	<0.001
Peptic ulcer disease, *n* (%)	361 (0.1)	134	<0.001
Peripheral vascular disorders, *n* (%)	77 424 (11.6)	29 253 (7.7)	<0.001
Psychoses, *n* (%)	25 649 (3.8)	16 322 (4.3)	<0.001
Pulmonary circulation disorders, *n* (%)	23 176 (3.5)	13 618 (3.6)	0.005
Renal failure, *n* (%)	141 311 (21.2)	59 066 (15.5)	<0.001
Rheumatoid arthritis/collagen vascular diseases, *n* (%)	23 187 (3.5)	14 150 (3.7)	<0.001
Valvular disease, *n* (%)	44 049 (6.6)	31 777 (8.3)	<0.001
Weight loss, *n* (%)	62 776 (9.4)	21 639 (5.7)	<0.001
Expected primary payor			
Medicaid, *n* (%)	11 718 (1.8)	4131 (1.1)	<0.001
Medicare, *n* (%)	589 302 (88.3)	350 750 (92.1)	<0.001
Private insurance, *n* (%)	54 309 (8.1)	19 849 (5.2)	<0.001
Self‐pay, *n* (%)	2023 (0.3)	896 (0.2)	<0.001
No charge, *n* (%)	164	69	0.036
Other, *n* (%)	8553 (1.3)	4381 (1.2)	<0.001
Missing, *n* (%)	1657 (0.2)	773 (0.2)	<0.001
Frailty index (mean ± SD)	1.42 ± 0.97	1.35 ± 0.91	<0.001
No. chronic conditions (mean ± SD)	1.88 ± 0.53	1.83 ± 0.51	<0.001
No. past fall‐related admissions (mean ± SD)	0.04 ± 0.21	0.06 ± 0.26	<0.001

## Results

### Population characteristics

Our population included patients who were admitted for fall‐ and non‐fall‐related injuries, as previously described.^26^ Compared with older adults admitted for other traumatic injuries (no fall group), those admitted for fall‐related injuries (fall group) were more likely to be women (67.9% *vs* 52.6%; *P* < 0.001) and aged ≥85 years (39.7% *vs* 20.1%; *P* < 0.001). They were less likely to be aged 65–74 years (22.4% *vs* 42.5%; *P* < 0.001). The proportion of older adults aged 75–84 years was the same between the groups (37.9% *vs* 37.4%; Table 1).

### Identifying factors associated with hospital admission for fall‐related injuries in older adults

#### LR model

**Figure 2:**
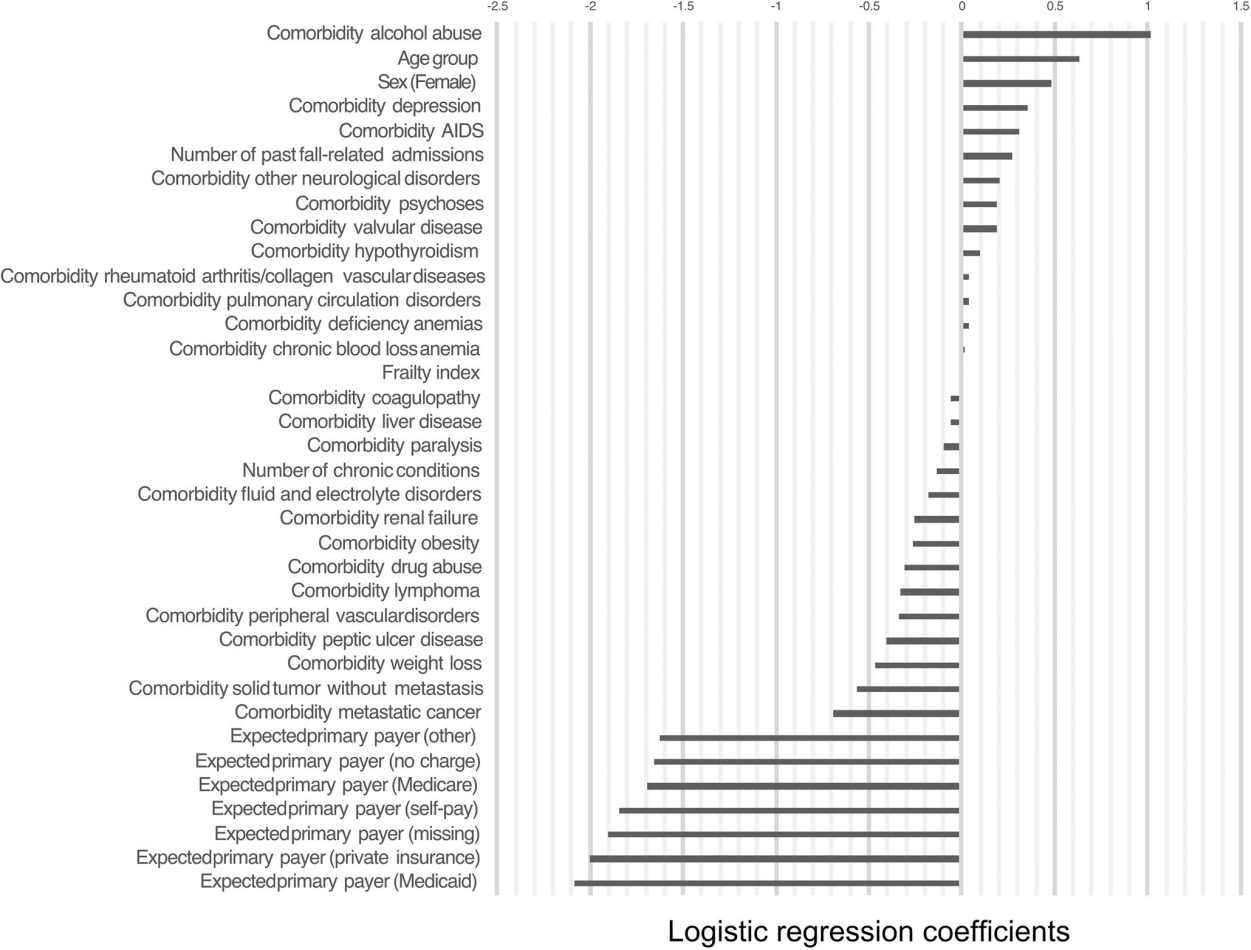
Variables associated with admission for fall‐related injuries based on the logistic regression model.

As shown in Fig. 2, alcohol abuse had the strongest association with admission for a fall‐related injury, followed by age. Being a woman or having been admitted for a fall injury in the past also put the patients at higher risk of being admitted for a fall injury. The frailty index was positively associated with hospital admissions for a fall injury. Using the LR model, admission for fall‐related injuries was independently associated with alcohol abuse (coefficient = 1.0124 [1–1.025], *P* < 0.001), age (coefficient = 0.629 [0.627–0.632], *P* < 0.001), being a women (coefficient = 0.480 [0.476–0.484], *P* < 0.001) and, to a lesser extent, frailty (coefficient = 0.007 [0.004–0.009], *P* < 0.001). The number of chronic conditions was inversely associated with admission for fall‐related injuries (coefficient = −1.135 [−0.130 to −0.139], *P* < 0.001). Older adults who had private insurance or Medicaid were less likely to be admitted for a fall‐injury (coefficient = −2.088 [−2.067 to −2.169] and −2.004 [−1.992 to −2.016], respectively, *P* < 0.001 for both; Table S4).

### RF model and predictive variable ranking

**Figure 3:**
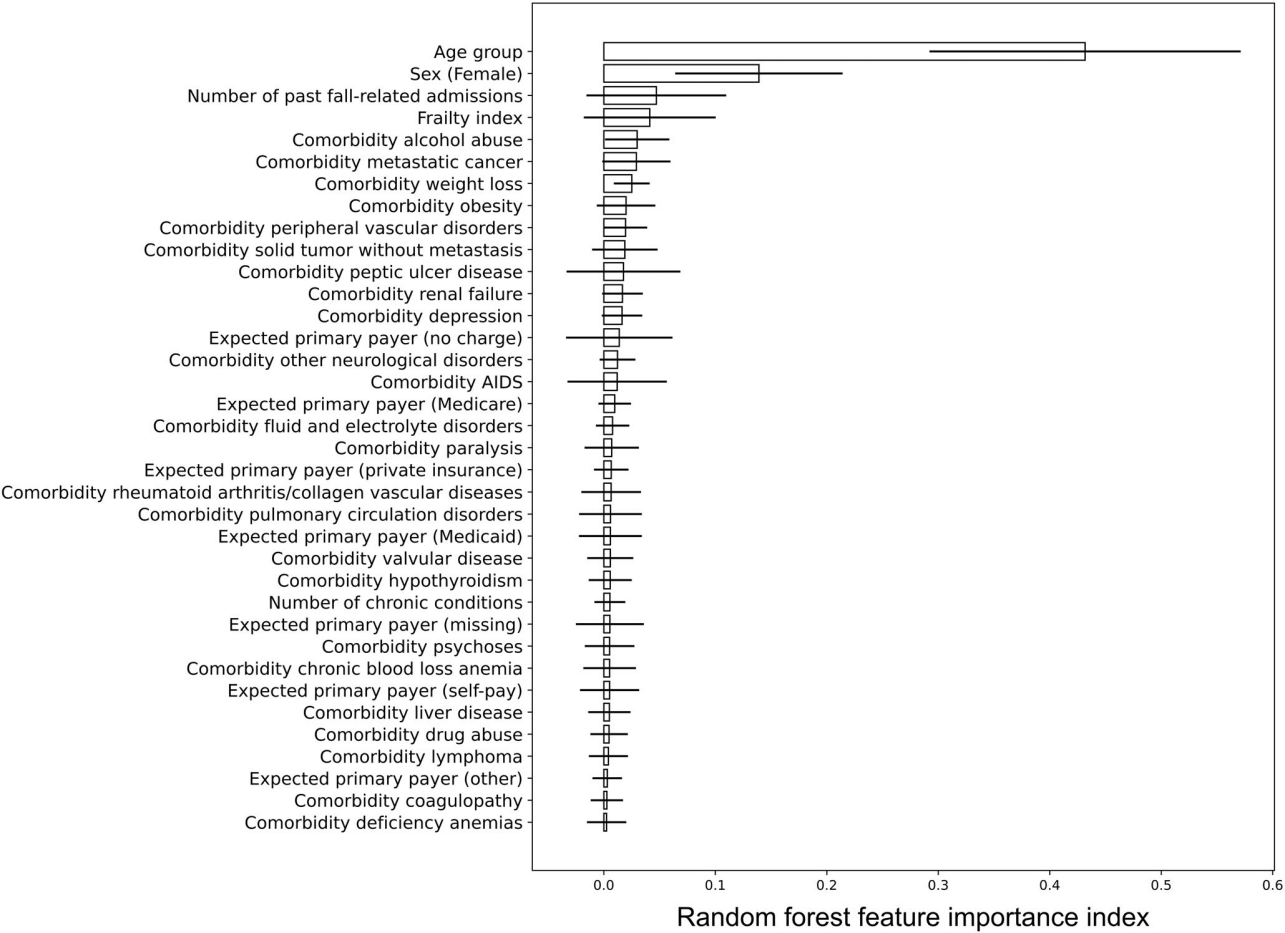
Predictive variable ranking based on the random forest model.

Using the RF model, the first four predictive variables for admission for fall‐related injuries in order of importance were age, frailty, being a woman and number of past fall‐related admissions. (Fig. 3).

### Partial dependence plot analysis

**Figure 4:**
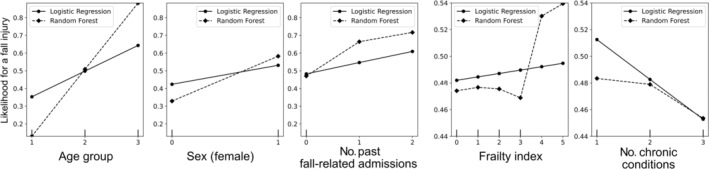
Partial dependence plots for the logistic regression and random forest models.

Partial importance plots showed that being older and/or a women put patients more at risk of admission for fall‐related injuries. Presenting frailty scores of ≥3 was highly associated with admission for fall‐related injuries, whereas the association decreased with the highest frailty scores. The lower the number of chronic conditions, the more at‐risk patients were for admission for fall‐related injuries. Patients with ≥11 chronic conditions (category 3) might be confined (home bound or bedridden), limiting their risk of fall and fall‐related injuries. (Fig. 4).

### Determining whether frailty can be used in combination with other factors as a predictor of admission for fall‐related injuries

The RF model was distilled into a single decision tree, with a maximum depth of the tree being 10. As shown in Table 2, 18 combinations of patient variables that led to a predicted fall admission were identified using this method. Being a women was one of the factors in the top nine combinations of factors associated with admission for fall‐related injuries. Being a woman and being aged >85 years were the two factors that appeared in the top five combinations. Being a man only presented in six of the 18 combinations. Frailty appeared in four of the 18 combinations. In combination 11, >80% (*n* = 18 658) of female patients aged 65–74 years with a frailty score >3, indicating that they were frail, were admitted for fall‐related injuries. In combination 17, 61.7% of women aged 65–74 years with frailty scores ≤3, but who had been previously admitted for fall‐related injury four or more times, were admitted for fall‐related injuries.

**Table 2:** Combinations of factors associated with an admission for fall‐related injuries

Gini index rank	High‐risk combination	No. negative cases	No. positive cases	Percentage of predicted positive cases Per combination	Percentage of combination's population over total population
1	Age ≥85 years **Female** Comorbidity weight loss: No Comorbidity metastatic cancer: No **Comorbidity peripheral vascular disorders: Yes** **Comorbidity renal failure: Yes**	8	17 711	100.0%	0.4%
2	Age ≥85 years **Female** Comorbidity weight loss: No Comorbidity metastatic cancer: No Comorbidity peripheral vascular disorders: No **Comorbidity obesity: Yes** **Comorbidity renal failure: Yes**	3	5143	99.9%	0.1%
3	Age ≥85 years **Sex: Female** Comorbidity weight loss: No **Comorbidity metastatic cancer: Yes**	20	6851	99.7%	0.2%
4	Age ≥85 years Male Comorbidity weight loss: No	1583	371 791	99.6%	8.4%
5	Age ≥85 years **Sex: Female** **Comorbidity weight loss: Yes**	787	66 436	98.8%	1.5%
6	Age 75–84 years **Sex: Female** Comorbidity weight loss: No Comorbidity metastatic cancer: No Comorbidity peripheral vascular disorders: No Comorbidity obesity: No	16 153	1 320 102	98.8%	30.1%
7	Age 65–74 years **Comorbidity alcohol abuse: Yes** **Sex: Female**	772	20 092	96.3%	0.5%
8	Age 75–84 years **Sex: Female** Comorbidity weight loss: No Comorbidity metastatic cancer: No **Comorbidity peripheral vascular disorders: Yes** Comorbidity renal failure: No Comorbidity obesity: No	3855	86 035	95.7%	2.0%
9	Age 75–84 years **Sex: Female** Comorbidity weight loss: No Comorbidity metastatic cancer: No Comorbidity peripheral vascular disorders: No **Comorbidity obesity: Yes** Comorbidity renal failure: No	2618	55 345	95.5%	1.3%
10	Age 75–84 years Male **Comorbidity alcohol abuse: Yes**	2714	20 878	88.5%	0.5%
11	Age 65–74 years Comorbidity alcohol abuse: No **Frailty index >3** **Female**	3669	14 989	80.3%	0.4%
12	Age 65–74 years **Comorbidity alcohol abuse: Yes** Sex: Male Comorbidity peripheral vascular disorders: No Comorbidity renal failure: No	11 125	31 278	73.8%	1.0%
13	Age ≥85 years Sex: Male **Comorbidity weight loss: Yes** Comorbidity fluid and electrolyte disorders: No	4475	12 532	73.7%	0.4%
14	Age 75–84 years **Sex: Female** **Comorbidity weight loss: Yes** Comorbidity fluid and electrolyte disorders: No Comorbidity renal failure: No	7065	16 845	70.5%	0.5%
15	Age 75–84 years Male Comorbidity alcohol abuse: No No. past fall‐related admissions: 0–3 **Comorbidity other neurological disorders: Yes** Comorbidity fluid and electrolyte disorders: No **Frailty index <2**	10 878	18 735	63.3%	0.7%
16	Age 75–84 years Male Comorbidity alcohol abuse: No No. past fall‐related admissions: ≥4	9991	16 097	61.7%	0.6%
17	Age 65–74 years Comorbidity alcohol abuse: No **Frailty index ≤3** Number of past fall‐related admissions: 4+ **Sex: Female**	13 156	14 695	52.8%	0.6%
18	Age 75–84 years Male Comorbidity alcohol abuse: No No. past fall‐related admissions: 0–3 Comorbidity other neurological disorders: No **Frailty index >3**	6420	6759	51.3%	0.3%

For each combination of variables, we reported the number of predicted positive and negative cases, the percentage of the positive cases for that population, and the percentage of the population over total trained samples.

In combination 15, 63.3% of men aged 75–84 years with frailty scores <2 and presenting with neurological disorders were admitted for fall‐related injuries. In combination 18, 51.3% of men aged 75–84 years with frailty scores >3 were admitted for fall‐related injuries.

## Discussion

In the present study, we investigated the ability of AI/ML models to predict hospital admission of older adults for fall‐related injuries by identifying nonclinical and clinical predictors. AI/ML models have been successfully used in other healthcare settings to develop preventative and/or curative protocols.^3^, ^4^, ^5^, ^6^ Using large datasets, such as the NRD, AI/ML models are programmed to learn a task; herein identifying predictors of hospital admission of older adults for fall‐related injuries. Only few studies have been published in the field.^9^, ^10^, ^37^ ML brings an ability to efficiently learn from multidimensional description indices (features) to provide outcome (admission‐for‐fall) predictions that are otherwise difficult to obtain using purely statistical approaches. As such, ML is typically considered a methodology of choice when diverse description data are available in sufficiently large numbers and the outcomes are known – so that well‐performing learning from data can be expected. Several choices in ML methods are made deliberately to enhance the interpretability of prediction results. For example, grouping numerical variables into categories can help clarify how demographic factors – such as age – affect the prediction of hospital admission for fall injuries, instead of relying on more granular, but less interpretable, data points. Normalization techniques, such as Z‐normalization, were not applied to retain the original numerical values. The mentioned techniques often improve predictive performance; however, they can negate interpretability. When it comes to machine learning interpretability, logistic regression is inherently interpretable, as its coefficients explain the influence of variables on outcomes. In contrast, RFs do not provide such an easy way to rank individual variables. Global methods, such as partial dependence plots and knowledge distillation, can describe the average behavior of any ML model, regardless of its complexity. In the present study, we summarized the RF model into a single decision tree, resulting in a more interpretable version of the original model.

Our approach yielded 18 models. Although frailty did not appear in the first 10 models, it did in four models. Interestingly, being a woman, aged 65–74 years with an mFI‐5 score >3 predicted admission for fall‐related injury in 80% of the population.

The first five models predicted admission for fall‐related injuries in the population aged ≥85 years presenting with or without comorbidities, such as renal failure, metastatic cancer, peripheral vascular disorder, obesity or weight loss. These results are consistent with the literature.^38^, ^39^, ^40^ Using Behavioral Risk Factor Surveillance System data, Moreland *et al*. showed that 33.8% and 13.9% of older adults aged ≥85 years reported a fall or a fall‐related injury with no difference between men and women.^38^ Grundstrom *et al*. showed that increased risk of falling in this population was associated with a decline of overall health status with age.^39^.

The next five models included three models that consider older women aged 75–84 years with or without comorbidities, one model including men aged 75–84 years with a history of alcohol abuse and one model including women aged 65–74‐years with history of alcohol abuse. Moreland *et al*. showed that the rate of older adults aged 75–84 years reporting at least one fall injury was higher than that reported by older adults aged 65–74 years.^38^ We previously showed that the rates of older adults aged 75–84 and ≥85 years admitted for a fall‐related injury were both approximately twice that of older adults aged 65–74 years old in the cohort used for the current study.^26^ Such difference in rates might have affected our AI/ML models in favor of the age categories the most represented in our dataset.

Finally, the two models including alcohol abuse as a predictor are in line with the literature. In fact, alcohol abuse has been shown to be associated with increased fall risk.^41^, ^42^

Model 11 predicts admission for fall injury in 80% of women aged 65–74 years with no history of alcohol abuse, but who are frail (frailty score >3). Although frailty is prevalent in women, it rises with age. It is estimated that 5–15% of women aged 65–74 years are considered frail.^43^ Frailty is a known risk factor for falls and has been used as a screening tool.^11^, ^18^ Despite using a quite different dataset, our data are in line with the literature, and support the need for fall prevention programs adapted to older adults at risk for falls. Lathouwers *et al*. showed that maintaining a mental, physical and socially active lifestyle was associated with a reduced risk of falling.^10^ In fact, it has been recommended that exercise, in the form of resistance (strength) training, and balance, gait and coordination training, should be included as part of multicomponent interventions to prevent falls in older people.^44^, ^45^, ^46^ For female patients in the 65–74 years age group, frailty could serve as an important screening tool for determining risk for falls. Additionally, patients in this age group might have the greatest ability to benefit from existing fall prevention programs or be the target population for new fall prevention programs developed in the future.

The present cohort study comes with limitations, starting with its retrospective nature. All findings are national estimates generated from the Nationwide Readmissions Database sample; the accuracy of the estimates and their associated variances depend on the weighting methods developed by the Agency for Healthcare Research and Quality. Additionally, the patient linkage numbers do not track the same patient across years of the NRD, and the hospital identifiers do not track the sampled hospitals across years of the NRD. Therefore, each year of the NRD was considered as a separate sample. An additional limitation is that the database is not positioned to assess patient medications, some of which are known to increase the risk of fall in older adults.^47^ Finally, the performance of the evaluated AI/ML models was upper‐bounded by the following factors: (i) the population (63.7%) was mostly patients admitted for non‐fall trauma‐related injuries, (ii) the number of studied variables was limited, and (iii) most variables were categorical. The present study did not consider or try to explain the effect of racial disparity or sex disparity on the predictive behaviors of ML models. The ML models used were chosen because, in our opinion, those models were appropriate for the study. Other models exist, such as SHAP, but there is not one perfect model. As the study was purposely carried out on selected variables, the interpretation of the results is limited to those variables. The generalizability of the findings, however, can also be limited by the retrospective nature of the data sources spanning from 2010 to 2014, which might not fully capture current trends or account for changes in healthcare practices and patient demographics in more recent years.

Despite these limitations, this study showed that AI/ML models can be utilized to identify populations at risk of admission for fall‐related injuries. Such tools could allow us to: (i) develop automated systems alerting providers regarding their patients' risk for fall‐related injuries, (ii) improve referrals to fall prevention programs, and (iii) better design prevention strategies adapted for each population, such as age‐adapted fall prevention programs.

Using AI/ML approaches, 18 signatures allowing us to identify older adults at risk of admission for fall‐related injuries were developed. Suh tools could be useful to healthcare providers in identifying older adults at risk for fall‐related injuries. Future studies using other databases, such as TQIP, are warranted to validate our high‐risk combination models.

## Funding

This research was supported by the University of Iowa Injury Prevention Research Center, and funded in part by grant # R49 CE002108‐05 of the National Center for Injury Prevention and Control/CDC.

## Disclosure statement

The authors declare no conflict of interest.

## Supporting information


**Data S1.** Supplementary Tables.

## Data Availability

The data that support the findings of this study are available from the corresponding author upon reasonable request.
