# Supplementary material for: Predicting admission for fall‐related injuries in older adults using artificial intelligence: A proof‐of‐concept study
Source: Geriatr Gerontol Int. 2025 Jan 12;25(2):232–42. doi: 10.1111/ggi.15066 (PMC11788240; doi:10.1111/ggi.15066)
Supplement: Supplementary file 1 — Data S1. Supplementary Tables. [file GGI-25-232-s001.docx]

**Supplementary Table 1**. Surrogate measures of impaired functional status. ICD-10, ICD-9 and corresponding NRD codes with their definitions are provided.
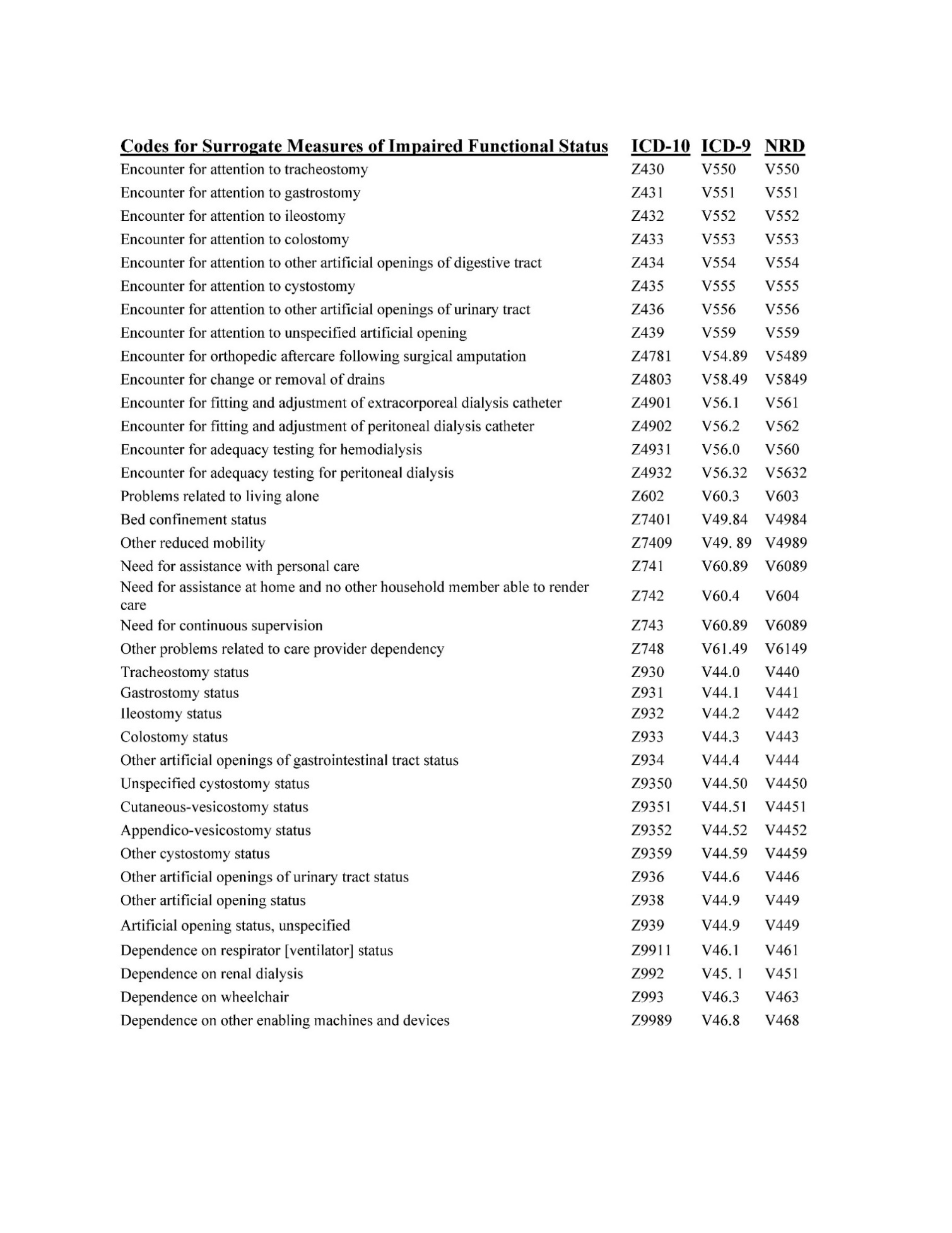


**Supplementary Table 2.** Descriptions of patient variables used, including the names of HCUP data elements and their descriptions.

| **Patient variables** | **Descriptions** |  | **Patient variables** | **Descriptions** |
| --- | --- | --- | --- | --- |
| **Patient baseline** | | | | |
| Age group | Ages in groups: 65-74, 75-84, and 85 years or older |  | Number of past fall-related admissions | Number of past hospital admissions for fall-related injuries |
| Sex (Female) | Indicates sex as female |  | Expected primary payer | Expected primary payer: 1-Medicare, 2-Medicaid, 3-private insurance, 4-self pay, 5-no charge, 6-other |
| **Comorbidities** | | | | |
| Comorbidity AIDS | Presented with acquired immune deficiency syndrome |  | Comorbidity metastatic cancer | Presented with metastatic cancer |
| Comorbidity alcohol abuse | Presented with alcohol abuse |  | Comorbidity other neurological disorders | Presented with other neurological disorders |
| Comorbidity deficiency anemias | Presented with Deficiency anemias |  | Comorbidity obesity | Presented with obesity |
| Comorbidity rheumatoid arthritis/collagen vascular diseases | Presented with Rheumatoid arthritis/collagen vascular diseases |  | Comorbidity paralysis | Presented with paralysis |
| Comorbidity chronic blood loss anemia | Presented with Chronic blood loss anemia |  | Comorbidity peripheral vascular disorders | Presented with Peripheral vascular disorders |
| Comorbidity coagulopathy | Presented with Coagulopathy |  | Comorbidity psychoses | Presented with Psychoses |
| Comorbidity depression | Presented with depression |  | Comorbidity pulmonary circulation disorders | Presented with Pulmonary circulation disorders |
| Comorbidity drug abuse | Presented with drug abuse |  | Comorbidity renal failure | Presented with Renal failure |
| Comorbidity hypothyroidism | Presented with Hypothyroidism |  | Comorbidity solid tumor without metastasis | Presented with Solid tumor without metastasis |
| Comorbidity liver disease | Presented with liver disease |  | Comorbidity peptic ulcer disease | Presented with Peptic ulcer disease excluding bleeding |
| Comorbidity lymphoma | Presented with lymphoma |  | Comorbidity valvular disease | Presented with Valvular disease |
| Comorbidity fluid and electrolyte disorders | Presented with Fluid and electrolyte disorders |  | Comorbidity weight loss | Presented with weight loss |
| Frailty index | 5-factor modified frailty index |  | Number of chronic conditions | Number of chronic conditions |

**Supplementary Table 3. Model performance in a 10-fold cross validation scheme with patient-level grouping.**

| **Classifier** | **Accuracy** | **std** | **Recall** | **std** | **Spec** | **std** | **Prec** | **std** | **F1** | **std** | **F0.5** | **std** | **F2** | **std** | **AUC ROC** | **std** |
| --- | --- | --- | --- | --- | --- | --- | --- | --- | --- | --- | --- | --- | --- | --- | --- | --- |
| **Logistic regression** | 0.645 | 0.001 | 0.644 | 0.001 | 0.646 | 0.001 | 0.516 | 0.001 | 0.573 | 0.001 | 0.537 | 0.001 | 0.613 | 0.001 | 0.693 | 0.001 |
| **Random forest** | 0.634 | 0.001 | 0.660 | 0.001 | 0.619 | 0.001 | 0.504 | 0.001 | 0.572 | 0.001 | 0.529 | 0.001 | 0.622 | 0.001 | 0.688 | 0.001 |
| **Naïve bayes** | 0.532 | 0.001 | 0.783 | 0.001 | 0.384 | 0.001 | 0.428 | 0.001 | 0.553 | 0.001 | 0.470 | 0.001 | 0.672 | 0.001 | 0.647 | 0.002 |
| **Linear SVM** | 0.672 | 0.001 | 0.350 | 0.001 | 0.862 | 0.001 | 0.597 | 0.002 | 0.441 | 0.002 | 0.523 | 0.002 | 0.382 | 0.001 | 0.689 | 0.001 |
| **Decision tree** | 0.649 | 0.002 | 0.622 | 0.006 | 0.665 | 0.006 | 0.522 | 0.002 | 0.567 | 0.002 | 0.539 | 0.001 | 0.599 | 0.004 | 0.701 | 0.001 |
| **Multi-layer perceptron** | 0.677 | 0.001 | 0.379 | 0.012 | 0.852 | 0.007 | 0.600 | 0.004 | 0.465 | 0.008 | 0.538 | 0.003 | 0.409 | 0.011 | n/a | n/a |

Std: Standard Deviation; SVM: support vector machine; Spec: Specificity; Prec: Precision

**Fbeta-measure** is a weighted harmonic mean of precision and recall with tunable parameter beta

F1 = F1 score, defined as the **Fbeta-measure** with a beta value of 1, is the balanced harmonic mean

F0.5 = F0.5 score, defined as the **Fbeta-measure** with a beta value of 0.5, emphasizes the importance of precision

F2 = F2 score, defined as the **Fbeta-measure** with a beta value of 2, emphasizes the importance of recall

**Supplementary Table 4. Logistic regression model coefficients and confidence intervals for variables associated with admission for fall-related injuries.**

| Variables | coefficient | std err | 95% CI | | Z score | p-value |
| --- | --- | --- | --- | --- | --- | --- |
|  |  |  | **Lower** | **Upper** |  | **P>\|z\|** |
| Alcohol abuse | 1.0124 | 0.006 | 1 | 1.025 | 159.912 | <0.001 |
| Age group | 0.6294 | 0.001 | 0.627 | 0.632 | 453.017 | <0.001 |
| Sex (Female) | 0.4799 | 0.002 | 0.476 | 0.484 | 216.943 | <0.001 |
| Depression | 0.3544 | 0.003 | 0.348 | 0.361 | 108.545 | <0.001 |
| AIDS | 0.3068 | 0.059 | 0.191 | 0.422 | 5.204 | <0.001 |
| Number of past fall-related admissions | 0.2681 | 0.004 | 0.26 | 0.277 | 61.101 | <0.001 |
| Other neurological disorders | 0.2068 | 0.003 | 0.201 | 0.213 | 66.829 | <0.001 |
| Psychoses | 0.1918 | 0.005 | 0.182 | 0.202 | 37.592 | <0.001 |
| Comorbidity valvular disease | 0.186 | 0.004 | 0.178 | 0.194 | 46.837 | <0.001 |
| Comorbidity hypothyroidism | 0.1017 | 0.003 | 0.096 | 0.107 | 36.78 | <0.001 |
| Comorbidity rheumatoid arthritis/collagen vascular diseases | 0.0388 | 0.005 | 0.028 | 0.049 | 7.177 | <0.001 |
| Comorbidity pulmonary circulation disorders | 0.0369 | 0.006 | 0.026 | 0.048 | 6.685 | <0.001 |
| Comorbidity deficiency anemias | 0.0347 | 0.002 | 0.03 | 0.04 | 13.894 | <0.001 |
| Comorbidity chronic blood loss anemia | 0.0125 | 0.008 | -0.004 | 0.029 | 1.513 | 0.13 |
| Frailty index | 0.0067 | 0.001 | 0.004 | 0.009 | 5.307 | <0.001 |
| Comorbidity coagulopathy | -0.0605 | 0.004 | -0.069 | -0.052 | -14.527 | <0.001 |
| Comorbidity liver disease | -0.0638 | 0.008 | -0.079 | -0.048 | -8.118 | <0.001 |
| Comorbidity paralysis | -0.0947 | 0.006 | -0.106 | -0.083 | -16.49 | <0.001 |
| Number of chronic conditions | -0.1345 | 0.003 | -0.139 | -0.13 | -53.404 | <0.001 |
| Comorbidity fluid and electrolyte disorders | -0.1777 | 0.002 | -0.182 | -0.173 | -78.493 | <0.001 |
| Comorbidity renal failure | -0.2589 | 0.003 | -0.265 | -0.253 | -90.994 | <0.001 |
| Comorbidity obesity | -0.2667 | 0.004 | -0.275 | -0.259 | -63.977 | <0.001 |
| Comorbidity drug abuse | -0.3071 | 0.012 | -0.33 | -0.284 | -26.427 | <0.001 |
| Comorbidity lymphoma | -0.3345 | 0.01 | -0.354 | -0.315 | -33.62 | <0.001 |
| Comorbidity peripheral vascular disorders | -0.3399 | 0.004 | -0.347 | -0.333 | -93.851 | <0.001 |
| Comorbidity peptic ulcer disease | -0.4098 | 0.053 | -0.514 | -0.306 | -7.711 | <0.001 |
| Comorbidity weight loss | -0.4677 | 0.004 | -0.476 | -0.46 | -114.931 | <0.001 |
| Comorbidity solid tumor without metastasis | -0.5607 | 0.006 | -0.573 | -0.548 | -86.868 | <0.001 |
| Comorbidity metastatic cancer | -0.6904 | 0.007 | -0.705 | -0.676 | -93.284 | <0.001 |
| Expected primary payer (other) | -1.622 | 0.01 | -1.642 | -1.602 | -161.943 | <0.001 |
| Expected primary payer (no charge) | -1.6543 | 0.072 | -1.795 | -1.514 | -23.026 | <0.001 |
| Expected primary payer (Medicare) | -1.694 | 0.005 | -1.704 | -1.684 | -340.56 | <0.001 |
| Expected primary payer (self-pay) | -1.8431 | 0.019 | -1.881 | -1.805 | -95.243 | <0.001 |
| Expected primary payer (missing) | -1.9028 | 0.025 | -1.952 | -1.853 | -75.021 | <0.001 |
| Expected primary payer (private insurance) | -2.0039 | 0.006 | -2.016 | -1.992 | -322.554 | <0.001 |
| Expected primary payer (Medicaid) | -2.088 | 0.011 | -2.109 | -2.067 | -198.345 | <0.001 |
